# Habitat Availability, Jurassic and Cretaceous Origins of the Deep‐Bodied Shark Morphotype and the Rise of Pelagic Sharks

**DOI:** 10.1002/ece3.72082

**Published:** 2025-08-29

**Authors:** Joel H. Gayford, Patrick L. Jambura, Julia Türtscher, Phillip C. Sternes, Scott G. Seamone, Kenshu Shimada

**Affiliations:** ^1^ College of Science and Engineering James Cook University Townsville Queensland Australia; ^2^ Shark Measurements London UK; ^3^ Department of Palaeontology, Faculty of Earth Sciences, Geography and Astronomy University of Vienna Vienna Austria; ^4^ Johannes Kepler University Linz Linz Austria; ^5^ Education and Conservation Department SeaWorld San Diego California USA; ^6^ Department of Marine Sciences Bahamas Agriculture and Marine Science Institute Andros Island Bahamas; ^7^ Department of Environmental Science and Studies DePaul University Chicago Illinois USA; ^8^ Department of Biological Sciences DePaul University Chicago Illinois USA; ^9^ Sternberg Museum of Natural History Fort Hays State University Hays Kansas USA; ^10^ Integrative Research Center Field Museum of Natural History Chicago Illinois USA

**Keywords:** adaptive evolution, diversification, Elasmobranchii, eustasy, morphology, niche partitioning

## Abstract

Macroevolutionary trends in vertebrate morphology fundamentally shape our understanding of marine ecosystems through deep time. Body form influences interactions between organisms and their environment, dictating their locomotor capabilities and ability to hunt/escape from other species. Sharks (Elasmobranchii: Selachii) have been suggested to broadly exhibit two discrete body forms: one ‘shallow‐bodied’ form associated with slow‐moving benthic species and a ‘deep‐bodied’ form typified by highly active pelagic taxa. Until now, no study has addressed the validity or evolution of these body forms in a phylogenetic framework. Hence, we lack understanding of when, why and how the body forms observed in extant species originally evolved. In this study, we reconstruct the evolutionary history of shark body form and provide statistical evidence to suggest three broadly discrete body forms among extant species. We find support for a benthic origin of sharks, with four discrete transitions to a pelagic‐type morphology occurring during the Jurassic and Cretaceous. Increased habitat availability during this time, driven by a combination of elevated sea temperature, eustatic sea level rise, continental fragmentation and diversification trends of actinopterygians and marine reptiles, could have facilitated the colonisation of the pelagic realm by Mesozoic sharks and the repeated independent evolution of body form consistent with extant pelagic species. We also propose that habitat availability and its taphonomic consequences may explain discordance between origination times suggested by molecular phylogenies and the fossil record.

## Graphical abstract

We find support for a benthic origin of sharks, with four discrete transitions towards pelagic‐type morphology occurring during the Jurassic and Cretaceous. Increased habitat availability driven by biotic and abiotic environmental change may have driven shifts towards pelagic‐type morphology. These results may help explain discordance between molecular and fossil‐based origination times.

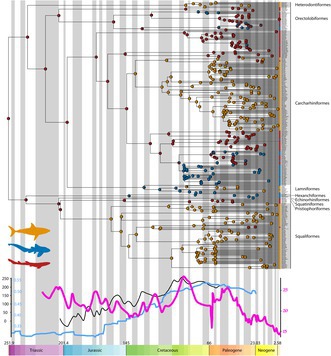

## 1 Introduction

Modern sharks (Elasmobranchii: Selachii) are thought to have originated at least 200 million years ago (Janvier and Pradel 2015). Despite substantial fluctuations in diversity, they remain a functionally important component of modern ecosystems from an ecological perspective (Gayford and Jambura 2025; Guinot and Cavin 2016, 2020; Ferretti et al. 2010; Heithaus et al. 2010; Janvier and Pradel 2015). The ecological diversity of extant sharks is substantial (Ferretti et al. 2010; Heithaus et al. 2010; Munroe et al. 2014), facilitated at least in part by morphological disparity (Gayford and Jambura 2025). Adult body size varies among sharks by ~8000% (Ebert et al. 2021), and various peculiar morphologies exist, such as the cephalofoil of hammerheads (Sphyrnidae) and the elongated caudal fin of thresher sharks (Alopiidae). Comparative phylogenetic studies have shown that morphological diversity and disparity in different components of shark morphology are associated with distinct patterns of ecological and phylogenetic signals (Bazzi et al. 2021; Gayford et al. 2024; López‐Romero et al. 2023; Sternes et al. 2024). Examples of such patterns include correlations between cranial and pectoral morphology with water depth and sea surface temperature, respectively (Gayford et al. 2024; Sternes et al. 2024). These correlations provide important insights into potential ecological drivers of trait evolution and morphological disparity and the extent to which these drivers may differ across different morphological features (Gayford et al. 2024).

Despite the highly specialised morphologies of some species, the body form of sharks is strongly conserved, with extant taxa falling into a small number of broad morphotypes delineated by ecological lifestyle. Early work by Thomson and Simanek (1977) suggested the presence of four principal body forms: the fast‐swimming pelagic morphotype, the generalised carcharhinid morphotype, the benthic morphotype, and the squalomorph morphotype. Subsequently, Sternes and Shimada (2020) performed a revised analysis of body form in sharks using a substantially enlarged dataset, confirming the close relationships between ecological lifestyle and body form (Wainwright 1991). However, this later study recovered only two morphotypes: (1) a shallow‐bodied shark with a more posteriorly placed first dorsal fin and a caudal fin with a more horizontal upper lobe and lower aspect ratio (span^2^/area) (Group A), and (2) a deep‐bodied shark with a more anteriorly placed first dorsal fin and a caudal fin with a more upright upper lobe and higher aspect ratio (Group B). Although exceptions do exist, the former of these two morphotypes consists primarily of benthic and benthopelagic taxa, whereas the latter contains the vast majority of pelagic sharks (Sternes and Shimada 2020). While these studies (Sternes and Shimada 2020; Thomson and Simanek 1977) were valuable to our understanding of morphological evolution in cartilaginous fishes and posed several hypotheses regarding the evolutionary history of shark body form, they did not utilise comparative phylogenetic methods. This represents a major limitation as phylogenetic non‐independence can influence perceived relationships between morphology and ecology. Moreover, a lack of empirical analyses to estimate ancestral states means that we have only limited understanding of how and when shark body forms have changed over geological time.

Throughout the Mesozoic and Cenozoic eras, genetic and fossil data indicate that the taxonomic diversity of sharks underwent substantial changes (Brée et al. 2022; Gayford and Jambura 2025; Guinot and Cavin 2016, 2020; Maisey et al. 2004). Similar shifts in diversity are observed in other marine vertebrate lineages during this time, including both actinopterygians and marine reptiles (Cavin et al. 2007; Guinot and Cavin 2016; Stubbs and Benton 2016). Faunal turnover of this nature is often linked, at least in part, to broad‐scale shifts in environmental conditions, including continental fragmentation, sea level and sea surface temperature (Cavin et al. 2007; Tennant et al. 2016; Zaffos et al. 2017). However, to date, no study has explicitly examined how these factors may have influenced the evolution of body form in sharks. Here, we estimate the timing of past evolutionary transitions in shark body form to better understand the potential evolutionary drivers of these shifts. We also test the supposed relationship between shark body form and ecological lifestyle (Sternes and Shimada 2020) using an empirical phylogenetic framework. We consider each of the three environmental parameters discussed above (eustatic sea level, continental fragmentation and sea surface temperature) as well as faunal turnover, and how they may have influenced the evolution of pelagic and benthic morphotypes observed in modern shark lineages, both directly and indirectly. These results not only improve our understanding of trait evolution and diversification in sharks but also provide insights into the eco‐evolutionary dynamics operating in past marine ecosystems.

## 2 Methodology

### 2.1 Data Collection

We extracted body form data for 452 extant shark species (including representatives from all orders) from Sternes and Shimada (2020). Sternes and Shimada (2020) used a geometric morphometric approach to study body form (including 13 fixed type II landmarks and 50 semilandmarks, cumulatively accounting for variation in the shape of the lateral body profile, the head, and fins), arguing for the presence of two broad categories of body form (Group A and Group B) on the basis of apparent clustering in morphospace. The authors noted that Group B contains most of the extant pelagic species, whereas benthic species comprised a far greater proportion of Group A (Sternes and Shimada 2020). However, no statistical analysis was performed to support the groupings adopted in this study. Hence, to eliminate the need for any a priori assumptions, we extracted raw principal component values (specifically the first 222 principal components, cumulatively explaining over 99.9% of the observed variance) for each species.

We also gathered ecological lifestyle data from Sharks of the World: A Complete Guide (Ebert et al. 2021), categorising species as either pelagic, benthic, or benthopelagic, following the approach of Sternes et al. (2024). We coded each species as ‘benthic’ based on habitat keywords of ‘benthic,’ ‘on muddy bottom,’ ‘on sediment,’ ‘bottom on insular continental shelves.’ Species were coded as ‘benthopelagic’ based on the key terms of ‘demersal,’ ‘near bottom’ or ‘near continental shelves.’ Species were coded as ‘pelagic’ based on the keywords of ‘pelagic,’ ‘epipelagic,’ ‘bathypelagic,’ ‘open ocean’ or ‘oceanic’ (note: functionally, water depth is not critical for classification of pelagic). This classification scheme is not free of limitations and is likely an oversimplification, where previous studies have identified over 20 shark ecomorphotypes (Compagno 1990; White et al. 2022). However, Sharks of the World (Ebert et al. 2021) is the most comprehensive and rigorous source of data regarding ecological lifestyle in extant sharks, comparable in scope and detail to the Fishbase database (Froese and Pauly 2010), which is commonly used as a source of ecological data for comparative phylogenetic studies of morphology. Moreover, Sharks of the World (Ebert et al. 2021) is standardised, as it has been compiled by the same authors and using fixed criteria, as opposed to a variety of sources as in Fishbase.

To assess the extent to which macroevolutionary shifts may be associated with shifts in habitat availability and global climate, we extracted data for Mesozoic and Cenozoic eustatic sea level, continental fragmentation and sea surface temperature from Haq (2014, 2018), Scotese et al. (2021) and Zaffos et al. (2017) respectively.

All analyses were based on the molecular phylogeny proposed by Stein et al. (2018). The original tree file contained 10,000 trees and 10 calibration points. We pruned each tree to match the taxa in our analyses (see above, and Table S1) using the *drop.tip* function in the R package *ape* (Paradis and Schliep 2019; R Core Team 2024). The pruned trees were then loaded into TreeAnnotator in Beast 2.7.1 (Drummond and Rambaut 2007) to infer a maximum clade credibility tree using the default settings (burn‐in percentage = 10; posterior probability limit = 0.0; node heights = common ancestor heights). The resulting tree contained 452 taxa of modern sharks (Selachii).

### 2.2 Data Analysis

All analyses were performed in the R statistical environment (R Core Team 2024).

To assess statistical support for discrete body form groupings (such as those proposed in Sternes and Shimada (2020)), we performed an optimal cluster analysis using the principal component data in the *nbclust* package (Charrad et al. 2014). We used a k‐means algorithm for clustering, grouping species into clusters such that the distance between data points and their associated cluster centre is minimised. We compared the fit of cluster alignments ranging from 2 to 10 clusters to determine the global optimum cluster alignment. The *nbclust* package utilises 30 different statistical indices (including the Dunn and Calinski‐Harabasz indices) to compare the fit of different cluster alignments and provides the optimal cluster alignment on the basis of the number of indices favouring any given alignment (Charrad et al. 2014).

Using the optimal cluster groupings (see Section 3) as a discrete variable, we calculated a generalised form of Blomberg's *K* statistic ‘$K_{\text{mult}}$’ (Adams 2014) to test the influence of phylogenetic non‐independence on body form in sharks using the *phylosig* function in the *phytools* package (Revell 2024). $K_{\text{mult}}$ values greater than 1 are associated with strong phylogenetic signal, whereas values lower than 1 suggest that phylogeny has little influence on observed trait distribution relative to Brownian Motion expectations (Adams 2014). This approach was chosen instead of multivariate measures of phylogenetic signal to avoid issues associated with the weighting of individual principal component variables.

To test for statistical associations between habitat and body form in sharks within a phylogenetic framework, we performed a series of phylogenetic logistic regressions in the *phylolm* package (Ho et al. 2016). This regression model is appropriate for testing for correlations between a binary response variable (in this case ecological lifestyle) and discrete and/or continuous predictor variables (in this case body form). *phylolm* uses maximum penalised likelihood estimates to determine regression coefficients. As ecological lifestyle consists of three categories, two separate models were fitted, one treating benthopelagic species as benthic and another treating them as pelagic. Fitting both of these models helps alleviate the intrinsic uncertainty associated with definitions of ‘benthopelagic’. Three additional phylogenetic logistic regression models were then fit, testing for correlation between body form and the presence/absence of each ecological lifestyle. Each of these models was compared to a null model (without covariates) on the basis of AIC values.

To estimate the timing and direction of past transitions in shark body form and habitat, we performed ancestral state reconstruction using a stochastic character mapping approach and assuming a Mk model of trait evolution. The Mk model describes a discrete k‐state Markov process in which evolutionary changes between discrete states can occur at any time, and the rate of change depends only on the current state and not on any previous states (Revell and Harmon 2022). We fit six different Mk models, three modelling the rate of change among body form groupings, and three modelling the rate of change among habitat groupings. In both cases, we fit the following models: an equal rates (ER) model that assumes that the transition rates between all pairs of states are equal, a symmetrical (SYM) model that assumes transition rates within state pairs are equal but can vary between different state pairs, and an ‘all rates different’ (ARD) model that does not assume any transition rates to be equal. We compared the fit of these models on the basis of AIC values.

To calculate the posterior probability of respective habitat and body form states at each node of the phylogeny, we finally simulated 10,000 stochastic character maps using our MCC phylogeny in the package *phytools* (Revell 2024). We assumed the model of trait evolution (either ER, SYM, or ARD) with the lowest AIC value and used a Bayesian approach to sample the transition matrix *q* from its prior distribution.

## 3 Results

### 3.1 Cluster Analysis

**FIGURE 1:**
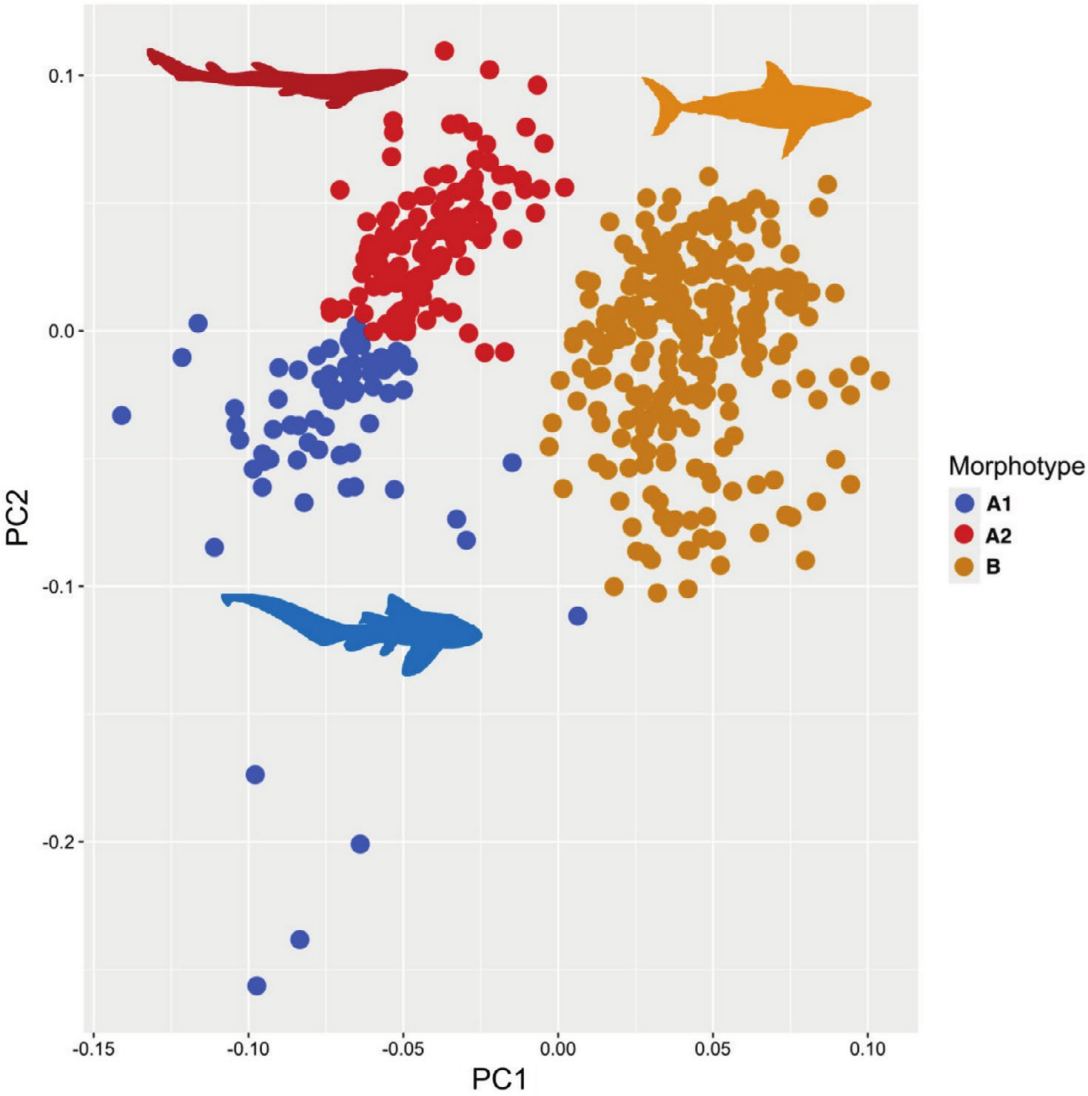
Plot showing shark body form morphospace occupation for all species included in the analysis. Species are coloured by the optimal body form category as recovered in the cluster analysis. Silhouettes are of representative species from each morphotype as follows: Orange (morphotype B, *Carcharhodon carcharias*), blue (morphotype A1, *Stegostoma tigrinum*), red (morphotype A2, *Scyliorhinus canicula* ).

K means cluster analysis indicated that the distribution of body form principal component data among extant shark species is best explained by a three‐category partition (Figure 1). These three categories broadly mirror the two body forms described by Sternes and Shimada (2020) and hence we name these categories as follows: Categories A1 and A2 (Figure 1) correspond to ‘Group A’ as described by Sternes and Shimada (2020), consisting of ‘shallow bodied’ species. These categories are both overwhelmingly represented by benthic taxa and can be distinguished by the relative aspect ratio of the caudal fin, as described in Sternes and Shimada (2020). Category B corresponds to ‘Group B’ as described by Sternes and Shimada (2020), consisting of ‘deep bodied’ species. Importantly, most pelagic species are found in this category. For a complete list of species in each category, please refer to the Table S1 associated with this article. Whilst the results of this cluster analysis are broadly consistent with the original results of Sternes and Shimada (2020), they are not identical. Besides the presence of three (rather than two) body form categories, our analysis also suggests that thresher sharks (*Alopias* spp.) fall in the A1 category (see Table S1), and not the B category (Group B) as initially proposed.

### 3.2 Phylogenetic Signal

The phylogenetic signal underlying body form variation in sharks was weaker than expected under Brownian Motion evolution ($K_{\text{mult}}$ = 0.151299, *p* < 0.096). Thus, phylogenetic non‐independence does not appear to be a significant determinant of body form variation among the species included in this study. This result does not support the notion of conservatism in shark body form, rather suggesting that similar body forms may have evolved convergently in multiple lineages.

### 3.3 Ecological Signal

Phylogenetic logistic regression (PLR) did not provide any support for body form being a significant predictor of the presence/absence of benthic or benthopelagic lifestyle (Table 1). However, the remaining three PLR models, including body form as a discrete covariate, did perform marginally better (ΔAIC > 2) than the null model. This indicates that there is a weak association between body form and ecological lifestyle, and particularly the pelagic lifestyle. Additionally, there is clearly one‐to‐many mapping of form to function (Wainwright et al. 2005) in shark body form, as evidenced by the deep‐bodied form of the predominantly benthic Heterodontiformes (Ebert et al. 2021). However, this does not prevent the evolution of certain body forms through deep time from being a necessary or sufficient precursor for subsequent shifts in the distribution and ecology of a given lineage (see below for further discussion in conjunction with shifts in the abundance of shallow marine environments).

**TABLE 1:** Phylogenetic logistic regression models provide weak support for a close relationship between shark body form and ecological lifestyle.

Covariate	AIC	Penalised log likelihood
None (null)	146.09	−71.49
Ecological lifestyle (benthopelagic = benthic)	142.54	−64.76
Ecological lifestyle (benthopelagic = pelagic)	142.54	−64.76
Presence or absence of pelagic lifestyle	142.54	−207.5
Presence or absence of benthic lifestyle	431.6	−64.76
Presence or absence of benthopelagic lifestyle	365.4	−175.3

*Note:* Body form and ecological lifestyle are coded as discrete variables (see Section 2).

### 3.4 Ancestral State Reconstruction

**FIGURE 2:**
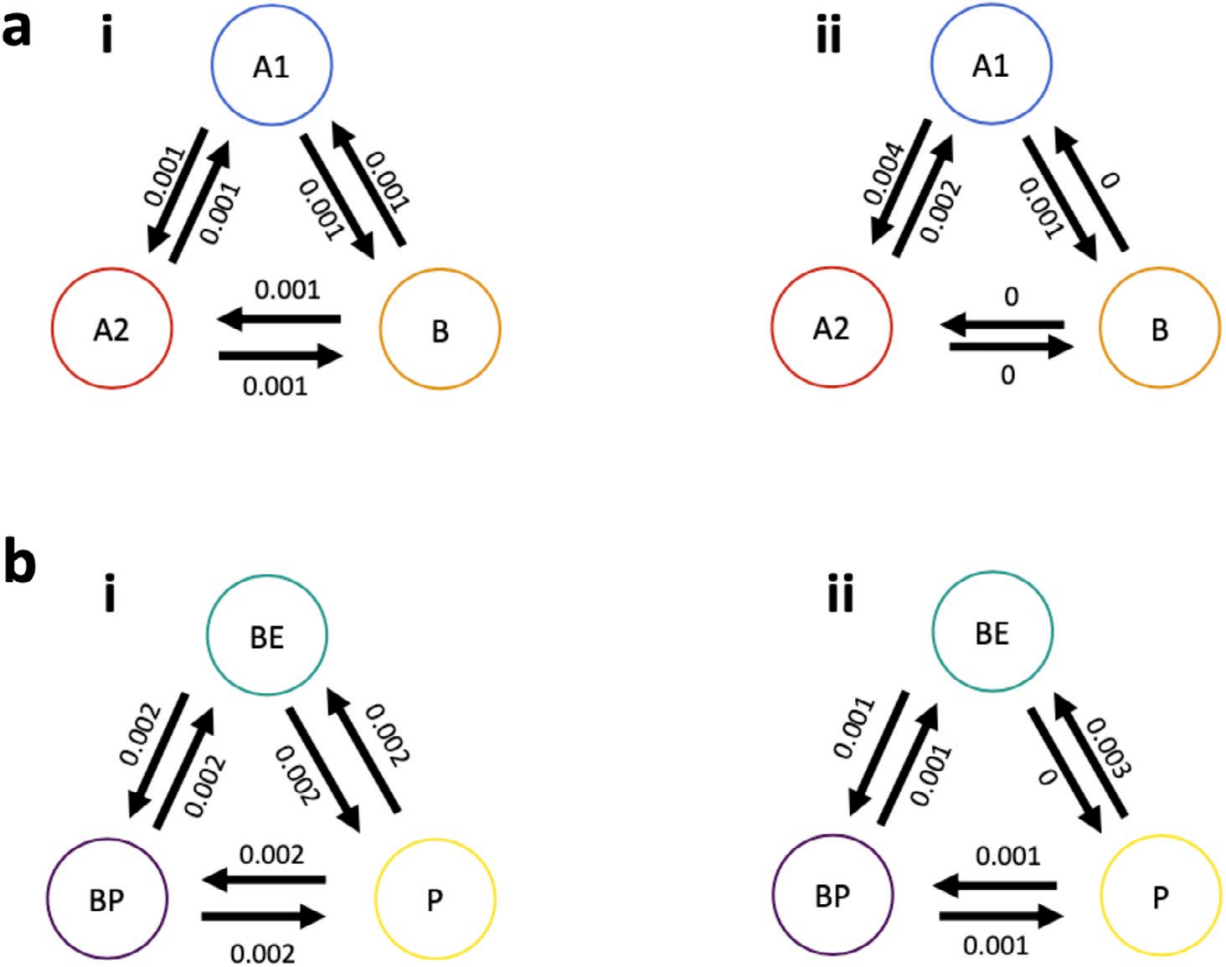
Visual representation of the ER (i) and ARD (ii) maximum likelihood estimates for the transition matrix, q, underlying models of body form (a) and habitat (b) evolution. Body form categories are labelled as per the cluster analysis, and habitat categories are labelled benthic (BE), benthopelagic (BP) and pelagic (P).

In the case of both habitat and body form, an ER (equal rates) transition matrix returned a better fit (dAIC > 2) model of discrete trait evolution than either ARD (all rates different) or SYM (symmetrical) matrices (Table 2). This indicates that transition rates between different habitat and body form categories are approximately equal through evolutionary time (Figure 2).

**TABLE 2:** Comparison of fit for Mk models of discrete character evolution for both body form and habitat, incorporating different varieties of transition matrices.

Discrete trait	Model	Log likelihood	AIC
Body form	ER	−156.5	314.91
Body form	ARD	−132.1	276.2969
Body form	SYM	−138.5	283.0637
Habitat	ER	−277.7	557.4081
Habitat	ARD	−239.0	489.9576
Habitat	SYM	−260.2	526.3594

**FIGURE 3:**
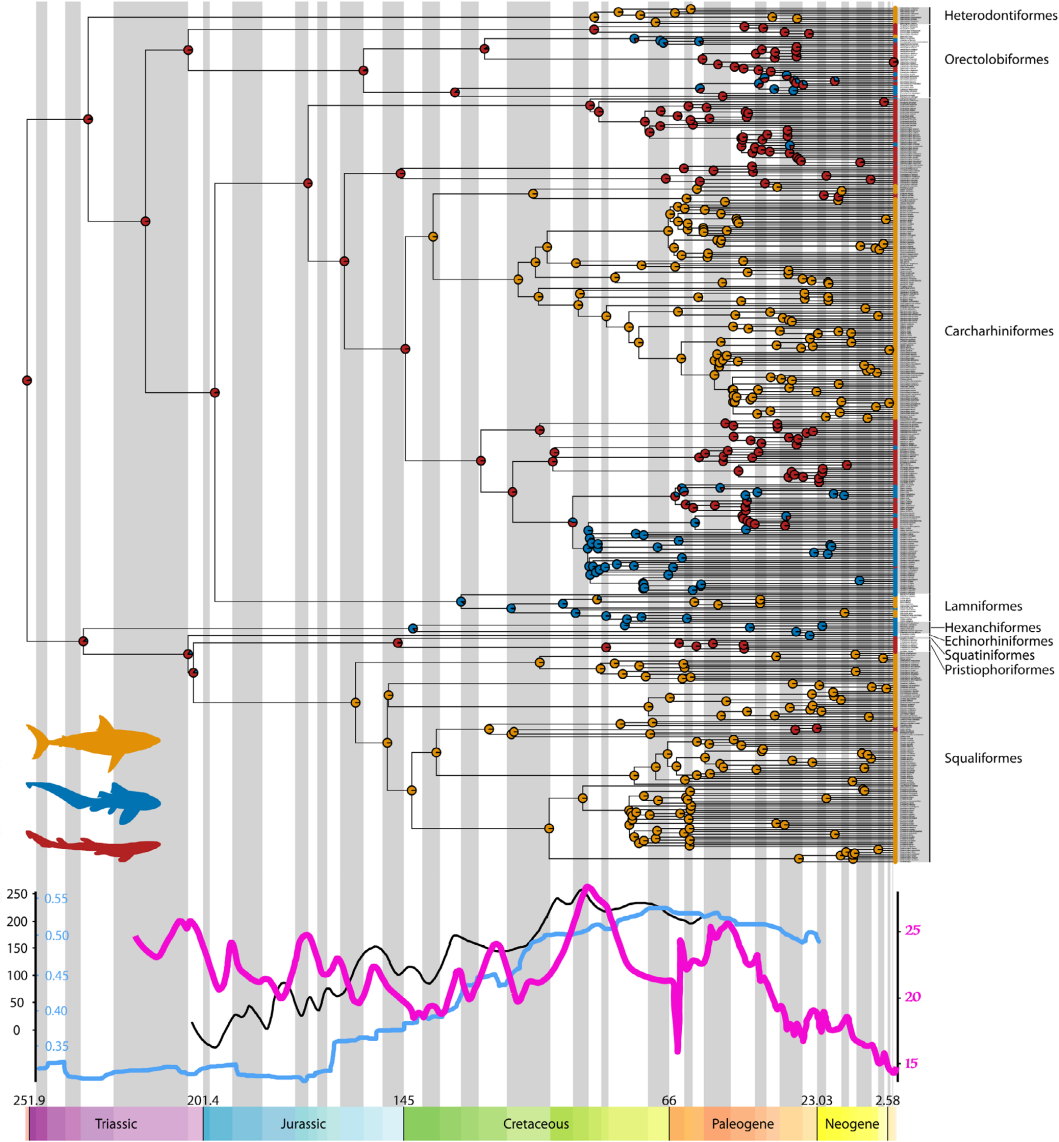
Ancestral state reconstruction showing evolutionary transitions in shark body form since the beginning of the Mesozoic, plotted against eustatic sea level (m, black line), continental fragmentation index (pale blue line) and sea surface temperature (°C, pink line). Coloured circles represent the probability of each body form at each node of the phylogeny, where orange represents morphotype B, and dark blue and red represent morphotypes A1 and A2, respectively. Silhouettes are of representative species from each morphotype as follows: Orange (morphotype B, *Carcharhodon carcharias*), blue (morphotype A1, *Stegostoma tigrinum*), red (morphotype A2, *Scyliorhinus canicula* ).

Ancestral state reconstruction of shark body form indicates that the common ancestor of all extant shark species exhibited a shallow‐bodied (A2) morphotype, and that four major independent transitions from shallow‐bodied morphotypes to the deep‐bodied (B) morphotype have occurred since the start of the Mesozoic: in Squaliformes during the Oxfordian (~163.5–157.3 Ma), in Lamniformes during the Coniacian (~89.8–86.3 Ma), in Carcharhiniformes during the Valanginian (~139.8–132.6 Ma) and in Heterodontiformes during the Coniacian (~89.1–86.3 Ma) (Figure 3). Most of these transitions occur during periods of pronounced increase in eustatic sea level (Haq 2014, 2018), a trend that also broadly coincides with periods of increased continental fragmentation (Figure 3). One outlier is Carcharhiniformes, which transitions to a deep‐bodied form during a period of relatively low eustatic sea level (Figure 3). Two reversions to a benthic morphotype (A2) from the pelagic morphotype (B) have subsequently occurred in Squaliformes and Carcharhiniformes, respectively, during the Cenozoic (Figure 3). At least seven independent transitions from the A2 morphotype to the A1 morphotype appear to have occurred, namely in Carcharhiniformes, Lamniformes, Squaliformes and Hexanchiformes. There appear to have been no transitions from the A1 morphotype to the A2 morphotype.

**FIGURE 4:**
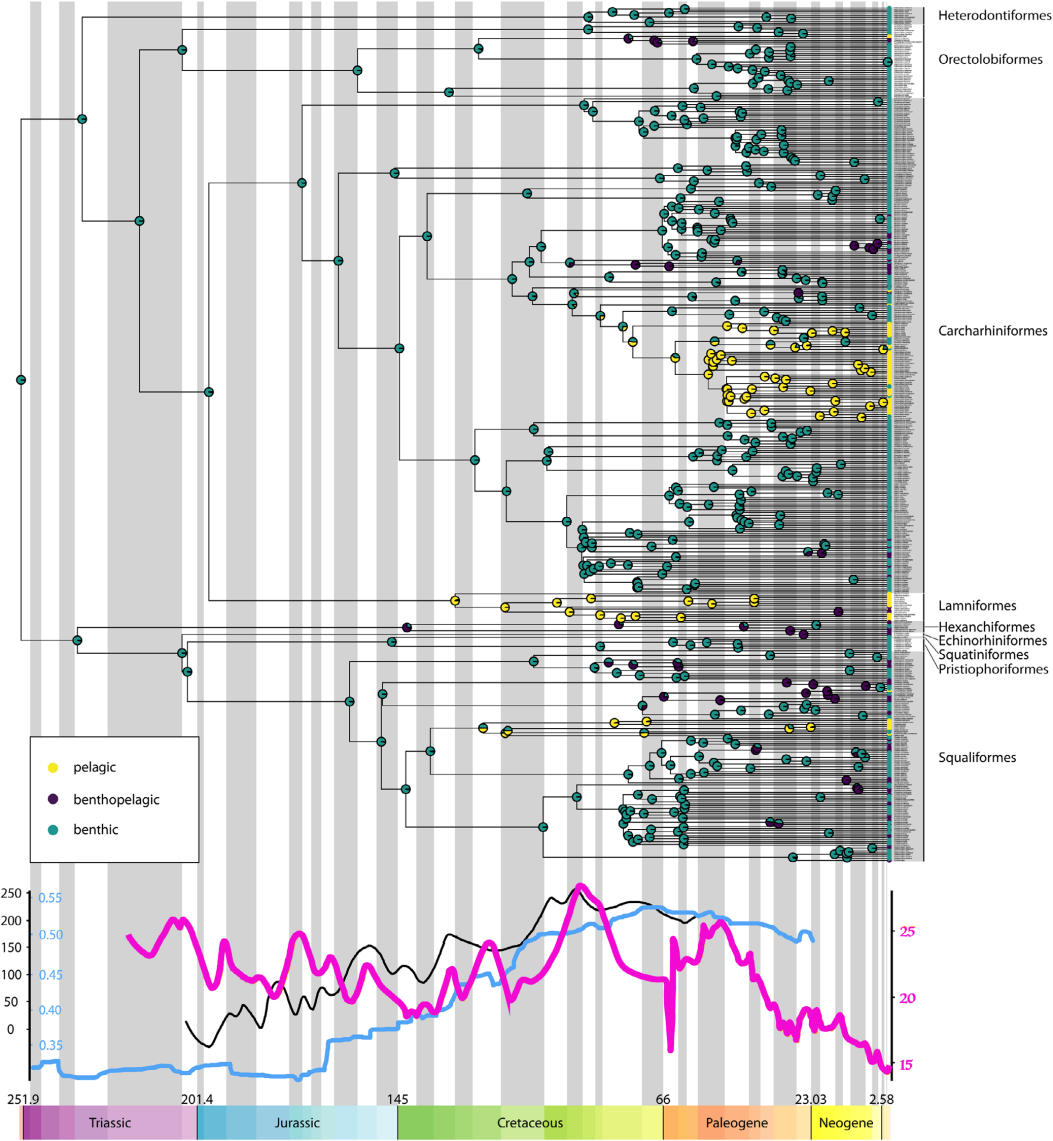
Ancestral state reconstruction showing evolutionary transitions in shark ecological lifestyle since the beginning of the Mesozoic, plotted against eustatic sea level (m, black line), continental fragmentation index (pale blue line) and sea surface temperature (°C, pink line). Coloured circles represent the probability of each ecological lifestyle at each node of the phylogeny, where yellow represents pelagic lifestyle, purple represents benthopelagic lifestyle and green represents benthic lifestyle.

Ancestral state reconstruction of shark ecological lifestyle indicates that the common ancestor of all extant shark species inhabited benthic environments with multiple subsequent transitions towards benthopelagic and pelagic environments (Figure 4). Only in Lamniformes do the estimated shifts in ecological lifestyle precede shifts towards pelagic‐type morphology, with ecological shifts occurring after morphological shifts in all other cases (Figures 3 and 4). Shifts in ecological lifestyle do often occur during periods of high eustatic sea level, sea surface temperature, and continental fragmentation (Figure 4), although there is no clear relationship (Figure 3).

## 4 Discussion

The purpose of this study was to provide a statistical test to uncover the number of body forms present among extant sharks, and then to elucidate the timing and nature of major shifts in body form occurring through shark phylogeny. Most significantly, we found that extant shark species fall into three discrete body form categories or morphotypes (Figure 1). The morphotype B is distinguished from the other two morphotypes as species in this category generally have deeper bodies as outlined in Sternes and Shimada (2020). This group also contains the majority of extant pelagic species, and is weakly associated with the presence or absence of a pelagic lifestyle (Table 1). The remaining two morphotypes (A1 and A2) are distinguished by the relative aspect ratio of the caudal fin and comprise overwhelmingly benthic species (see Table S1). These morphotypes bear broad resemblance to the two categories laid out in Sternes and Shimada (2020) but do exhibit several differences as outlined in the results. Herein, we refer to morphotype B as the pelagic (deep‐bodied) body form and morphotypes A1 and A2 as benthic (shallow‐bodied) body forms, as the former contains most extant pelagic species, and the latter two morphotypes are comprised predominantly of benthic species.

Our results indicate that although body form is generally conserved across modern shark species, at least four major independent shifts towards pelagic body form (i.e., morphotype B) have occurred in the last 200 million years (Figure 3). A number of reversions from pelagic to benthic body forms (A1 and A2) have occurred, as have shifts between the two shallow‐bodied benthic morphotypes (Figure 3). Notably, three of the four shifts towards pelagic‐type body forms appear to coincide with periods of eustatic sea level increase and increased continental fragmentation (Figure 3). Maximum likelihood estimates for the transition matrix underlying body form evolution indicate no difference in rates of reversion among groups (Figure 2), although our ancestral state reconstruction analyses suggest that transitions from the ancestral body form (A2) to the other benthic form (A1) are far more frequent than the reverse scenario (Figure 3). Here, we consider the evidence for four putative biotic and abiotic drivers of these shifts in body form, comparing our results to taxonomic and morphological diversification trends in other marine vertebrate lineages and drivers of global environmental change. We then synthesise this information to formulate a hypothesis for the timing and nature of each of the four major shifts in shark body form.

### 4.1 Interactions Between Sharks and Mesozoic Marine Reptiles

Reduced ecological interactions between sharks and Mesozoic marine reptiles driven by eustatic sea level changes provide one potential explanation for multiple independent transitions towards the modern pelagic shark morphotype. Mesozoic marine reptiles comprised several radiations including Ichthyopterygia (251–90 Ma), Plesiosauria (203–66 Ma) and Mosasauria (121–66 Ma) that underwent substantial shifts in distribution and diversity prior to their extinction (Bardet 1994; Motani 2009). The exact nature of ecological interactions between sharks and marine reptiles in Mesozoic ecosystems remains unknown. However, the hypothesised trophic position of large‐bodied marine reptiles (Fröbisch et al. 2013; Polcyn et al. 2014; Sachs et al. 2023) and apparent convergence with the pelagic shark morphotype (at least for ichthyosaurs and mosasaurs) (Lindgren et al. 2010; Lingham‐Soliar 2016; Motani and Shimada 2023) would indicate that both predatory and competitive interactions are plausible. Indeed, these relationships may also have been bidirectional, as Mesozoic sharks and marine reptiles were both represented by large ‘apex predator’ taxa as well as smaller‐bodied forms that may have been predated upon by the former (Everhart 2004; Rothschild et al. 2005; Schulp et al. 2013; Shimada 1997). Whilst some Mesozoic marine reptiles such as certain ichthyosaur and mosasaur taxa were possibly capable of deep diving (Humphries and Ruxton 2004; Motani et al. 1999; Schulp et al. 2013), they were predominantly restricted to the surface waters as air breathers and would have likely had comparatively little influence over deep‐water benthic and benthopelagic shark species.

Our results indicate that prior to the Late Jurassic, all sharks exhibited benthic, shallow‐bodied morphotypes and likely inhabited the benthic realm (Figures 3 and 4). This is largely in accordance with the fossil record because all known Jurassic shark taxa are thought to be benthic with the possible exception of *Sphenodus* (Thies and Reif 1985). During this time, eustatic sea level did not exceed 100 m above that of modern‐day oceans (Figure 3; Haq 2014, 2018). Eustatic sea level rise occurring during the Oxfordian, Hauterivian, and mid‐Cretaceous (Haq 2014, 2018) could have increased the proportion of pelagic environments in the water column that were largely inaccessible to marine reptiles. This in turn could have created vacant niches in these newly added deeper pelagic environments, facilitating the radiation of previously benthic lineages and favouring the evolution of the more ichthyosaur‐like pelagic shark morphotype. Moreover, these shifts in body form also coincide with the decline and eventual extinction of the ichthyosaurs (Motani 2009; Stubbs and Benton 2016), which could have further contributed to increased pelagic niche availability. However, the decline of ichthyosaurs alone is not sufficient to explain shifts in shark body form, as many ichthyosaurs did not leave vacant niches but were presumably excluded competitively by plesiosaurs (Sachs et al. 2023; Stubbs and Benton 2016).

### 4.2 Diversification of Actinopterygii

The ecological diversification of actinopterygian fishes may also have offered ecological opportunities that facilitated the evolution of the modern pelagic shark morphotype. The same factors discussed above (increased eustatic sea level and the demise of Ichthyopterygia) could have also favoured migration of benthic actinopterygian lineages into newly expanded deeper pelagic habitats through reduced competition and predation. Diversification analyses considering both Actinopterygii and Chondrichthyes suggest that increases in ‘fish’ taxonomic richness occurring over the Jurassic result from gradual ecological turnover and opportunistic radiation into vacant niches (Friedman and Sallan 2012; Gayford and Jambura 2025; Kriwet et al. 2009). Actinopterygian taxa represent a major prey source of modern sharks (Heithaus and Vaudo 2004), a relationship that has likely persisted through geological time (Fanti et al. 2016; Maisey 1994). Hence, the radiation of actinopterygian fishes into the widened pelagic zone as oceans deepened may have further increased the ecological opportunity available to Mesozoic sharks. There is also circumstantial evidence for competition between actinopterygians and chondrichthyans shaping the diversity of both lineages. For example, the decline in the functional diversity of sharks appears to be associated with niche replacement by actinopterygians (Cooper and Pimiento 2024; Whitenack et al. 2022).

There are additional factors that may have favoured a pelagic lifestyle in actinopterygian taxa during the Jurassic–Cretaceous interval accompanied by shifts towards a more pelagic morphotype in sharks. Among these factors are whole genome duplication and the evolution of oxygen‐secreting swim bladders, both of which played key roles in the morphological and ecological diversification of marine actinopterygians (Berenbrink et al. 2005; Hurley et al. 2007). While phylogenetic uncertainty has prevented consensus over the exact timing of these key evolutionary events (Davesne et al. 2021), they may both have occurred during the Jurassic–Cretaceous interval (Berenbrink et al. 2005; Hurley et al. 2007) in line with shifts in shark body form (Figure 3). Moreover, the latter of these innovations is intrinsically linked to pelagic environments given that it is the swim bladder that provides buoyancy control in osteichthyan fishes (Alexander 1982). These lines of reasoning lead us to suggest that during the interval across which the modern pelagic shark morphotype arose, actinopterygian prey sources in pelagic environments likely increased in diversity, abundance, and that this resulting ecological opportunity could have partially facilitated transitions towards the pelagic morphotype.

### 4.3 Continental Fragmentation, Sea Level Rise and Shallow Benthic Environments

The increasing availability of shallow benthic environments during the Jurassic–Cretaceous interval may also have facilitated the evolution of novel shark morphotypes. Substantial continental fragmentation and eustatic sea level rise occurred during this interval, both of which resulted in the creation of abundant warm shallow seaways (Haq 2014, 2018; Scotese et al. 2021). Our results suggest that the timing of three transitions towards the modern pelagic shark morphotype occurred in conjunction with both eustatic sea level rise and continental fragmentation (Figure 3). It is unlikely that sharks would have initially diversified into emerging pelagic environments within these shallow ecosystems due to the abundance of competing or predatory marine reptiles (including ichthyosaurs, plesiosaurs and thalattosuchians) at this time (Stubbs and Benton 2016). However, it is possible that radiation into shallow benthic environments as a result of continental fragmentation and sea level rise could have indirectly facilitated adaptation for life in the pelagic realm. These shallow benthic environments, particularly coral reefs, are associated with elevated speciation rates in sharks and other marine lineages (Gayford and Jambura 2025; Sorenson et al. 2014). It may be the case that character transitions associated with shifts from benthic to pelagic morphology in extant species were initially driven by some other selective pressure in shallow benthic environments, with the pelagic morphotype later co‐opted for life out of the benthos following the decline of other pelagic predators. Evidence for this one‐to‐many mapping of form to function (Wainwright et al. 2005) comes from the fact that Heterodontiformes, a primarily benthic lineage, exhibit the pelagic morphotype (Sternes and Shimada 2020), with the reverse case being presented by *Alopias* spp. (Figure 1). Indeed, many ostensibly pelagic carcharhinid sharks also inhabit relatively shallow coastal environments (Ebert et al. 2021), demonstrating that the pelagic morphotype performs well not only in true pelagic oceanic settings. Furthermore, our results indicate that transitions from benthic morphotypes to the pelagic morphotype generally (with Lamniformes being the exception) precede ecological transitions from benthic to pelagic lifestyles (Figures 3 and 4). Hence, morphology consistent with the pelagic morphotype likely evolved initially in a benthic setting and later facilitated expansion into pelagic environments in most modern pelagic lineages.

### 4.4 Temperature

The fossil record indicates that temperature has been a critical factor for shark evolution as shark diversity increases during periods of warming, whereas shark diversity declines during periods of cooling (Brée et al. 2022; Condamine et al. 2019; Cooper and Pimiento 2024; Gayford and Jambura 2025; Guinot and Cavin 2020; Guinot and Condamine 2023). Similar trends are observed in other marine lineages, with multiple actinopterygian radiations linked to high sea surface temperatures (Cavin et al. 2007; Guinot and Cavin 2020). A recent study has shown that warm sea surface temperatures, along with ocean anoxic events, may have driven some benthic sharks into the pelagic zone, and once in the pelagic zone, sharks exhibited adaptive evolution in their pectoral fins, a key morphological structure for locomotion (Sternes et al. 2024). Similarly, our results indicate that several shifts from the benthic to pelagic morphotype occurred during time periods in which sea surface temperatures were much higher than in modern oceans (Figure 3), although the transitions themselves do not occur during global or local sea surface temperature optima. It is worth noting that temperature does not act in isolation, and rising ocean temperatures result in the melting of ice caps and subsequent rise in sea levels. Indeed, it is practically impossible to decouple the effects of the expansion of shallow benthic regimes due to continental fragmentation from rising ocean temperatures on the evolution of marine life. Nevertheless, ocean temperature has likely been of great importance to the evolution of pelagic shark morphology, whether directly or indirectly.

### 4.5 Synthesis

Ultimately, each of the four factors discussed above implicates habitat availability, either directly or indirectly, as the major driving force of body form evolution in sharks. Our results suggest discordance between the evolution of pelagic‐type morphology and occupancy of pelagic environments (Figures 3 and 4), and thus the evolution of pelagic sharks can be separated into two broad evolutionary ‘events’: the acquisition of the pelagic morphotype and the colonisation of pelagic environments. We hypothesise that both stages of pelagic shark evolution depended critically on the availability of specific habitat types, in turn driven by eustatic sea level rise, continental fragmentation, changes to sea surface temperature, and the composition of marine communities. Eustatic sea level rise (in part driven by elevated sea surface temperature) and continental fragmentation directly contributed to increased habitat availability during the Jurassic and Cretaceous (Haq 2014, 2018), in demersal and shallow coastal zones, respectively. This increased habitat availability could have resulted in the evolution of the pelagic shark morphotype either due to hydrodynamic differences between these environments and the deep benthos or due to selective pressures relating to the capture and handling of new actinopterygian prey species radiating into these environments at a similar time (Cavin et al. 2007). A third potential explanation, in the case of shallow coastal environments, is that species diversification resulting from increased habitat availability (Mull et al. 2022) increased the need for niche partitioning among benthic shark species, consequently driving the evolution of novel, pelagic‐type morphology. This mechanism of selection may well explain the latter body form transitions observed in Carcharhiniformes and Heterodontiformes (Figure 3) but is insufficient to explain the first two origins of the pelagic morphotype in the Late Jurassic (Figure 3), a period of perceived stasis in chondrichthyan diversification (Kriwet et al. 2009). The subsequent demise of the ichthyosaurs during the Cretaceous (Bardet 1994) would then have facilitated colonisation of the pelagic realm by demersal and coastal lineages that had evolved pelagic‐type morphology. More broadly, the availability of shallow coastal environments has been a key driver of several major diversification events among neoselachians (Gayford and Jambura 2025; Sorenson et al. 2014).

### 4.6 Congruence and Consilience Between Molecular Phylogenies and the Fossil Record

One of the curious paradoxes in the evolutionary history of sharks is the frequent lack of congruence between the fossil occurrence data and origination times calibrated from reconstructed phylogenies. Despite a near‐total lack of corroborating fossil evidence in many instances, many modern shark clades are suggested to have emerged during the Mesozoic on the basis of molecular phylogenetic analyses (Heinicke et al. 2009; Marion et al. 2024; Martin et al. 2002; Sorenson et al. 2014; Stein et al. 2018) and comparative morphological studies (e.g., Shirai 1996). One possible explanation for this discrepancy is that Mesozoic members of these clades may not yet have been recognised as their ancestors. The fossil record of sharks overwhelmingly consists of isolated teeth, and if ancestral taxa lack the apomorphic dental characters of extant (or well‐documented extinct) representatives, this can both hamper the recognition of these taxa's presence in the fossil record and result in underestimation of their first occurrences (Maisey et al. 2004; Shimada et al. 2015). Although the rate of molecular evolution in sharks is known to be generally slower compared to mammals (Martin et al. 2002; Sendell‐Price et al. 2023), it has been suggested that the rates of morphological and molecular evolution may not have been synchronous across different shark taxa (Stone and Shimada 2019).

Besides possible asynchronous evolution between morphology and molecules, our present study also offers another plausible explanation for the observed differences between the morpholoy‐based fossil record and molecular‐based origination times. Most notably, the prevalence of benthic environments with a limited pelagic zone prior to major sea level rise and continental fragmentation in the Late Cretaceous (and the predominant occurrence of sharks within these environments, see Figure 4) implies that the position of the shorelines (and thus depositional environments for fossilisation) before this time would likely have extended well beyond the edge of the present‐day continental shelves developed during the Pleistocene (Cappetta 2012). Thus, the scant fossil record of Mesozoic sharks before the Late Cretaceous (Harris and Macmillan‐Lawler 2016; Maisey 2012) may simply result from pre‐Late Cretaceous Mesozoic marine rocks forming largely in isolation from the modern continental landmasses from which the majority of the fossil record is drawn. This taphonomic bias not only would explain the apparent scarcity of pre‐Late Cretaceous Mesozoic shark remains but also would account for the pre‐Cenozoic origination times of many shark clades from molecular data that have until now seemingly been overestimated. Our results, indicating that prior to the Jurassic–Cretaceous interval, sharks were restricted to deep, benthic environments due to their body form, biotic interactions with other lineages and biogeographical factors, have impactful consequences on our understanding of shark diversification dynamics and the fossil record.

## 5 Limitations

All macroevolutionary analyses are subject to a number of limitations, which provide essential context through which results must be considered. In the case of this study, one limitation is the use of scientific illustrations in lieu of morphological data from real specimens. Whilst the accuracy and suitability of these illustrations for such research is supported by geometric morphometric analyses (Siders et al. 2023), they do necessitate the omission of intraspecific variation. Consequently, the degree of variation observed in our results may be an oversimplification. A further potential simplification derives from the choice of macroevolutionary models. In this study, we used the classical Mk model of trait evolution. While this is a suitable model for the analyses presented, more complex models exist that account for heterogeneity in rates of evolution and underlying adaptive regimes (e.g., Beaulieu et al. 2017). Finally, the choice of phylogeny used in comparative analyses can impact the results. In this study, we used the polytomy‐resolved phylogeny presented in Stein et al. (2018), which is subject to the limitations associated with birth‐death polytomer resolver models.

## 6 Conclusions

It is likely that no single factor drove the evolution of the pelagic shark morphotype to the exclusion of all others, where eustatic sea level, continental fragmentation, the demise of marine reptiles, diversification of actinopterygians, and elevated sea surface temperature all played some role, either directly or indirectly, in the evolution of pelagic‐type morphology and subsequent colonisation of the pelagic realm. Regardless of the specific drivers, basal sharks were benthic in nature (Figure 4; Sternes et al. 2024), and the pelagic morphotype has evolved independently on four occasions during the Late Jurassic and Cretaceous (Figure 3), three of which preceded colonisation of pelagic environments (Figure 4). These findings provide important insight into the timing of major morphological and ecological transitions occurring in Mesozoic marine ecosystems and should form the basis of future work incorporating additional information from the fossil record alongside further palaeoclimatic studies. The relationships uncovered between body form and habitat availability also shed new light on patterns of discordance between molecular phylogenies and the fossil record, potentially contributing to the future resolution of phylogenetic uncertainty within the selachian crown group.

## Author Contributions

**Joel H. Gayford:** conceptualization (lead), formal analysis (lead), writing – original draft (lead), writing – review and editing (equal). **Patrick L. Jambura:** conceptualization (equal), writing – original draft (equal), writing – review and editing (equal). **Julia Türtscher:** conceptualization (equal), writing – original draft (equal), writing – review and editing (equal). **Phillip C. Sternes:** conceptualization (equal), data curation (equal), writing – original draft (equal), writing – review and editing (equal). **Scott G. Seamone:** conceptualization (equal), writing – original draft (equal), writing – review and editing (equal). **Kenshu Shimada:** conceptualization (equal), writing – review and editing (equal).

## Conflicts of Interest

The authors declare no conflicts of interest.

## Supporting information


**Table S1:** Morphotype classifications for all 452 species included in this study, as recovered by K means cluster analysis.

## Data Availability

No new datasets were developed for this study. Details of primary literature sources for morphological, environmental and phylogenetic data are presented within the methodology.
