# Supplementary material for: Habitat Availability, Jurassic and Cretaceous Origins of the Deep‐Bodied Shark Morphotype and the Rise of Pelagic Sharks
Source: Ecol Evol. 2025 Aug 29;15(9):e72082. doi: 10.1002/ece3.72082 (PMC12396820; doi:10.1002/ece3.72082)
Supplement: Supplementary file 1 — Table S1: Morphotype classifications for all 452 species included in this study, as recovered by K means cluster analysis. [file ECE3-15-e72082-s001.docx]

**Table S1: Morphotype classifications for all 452 species included in this study, as recovered by K means cluster analysis**

| **Species** | **Order** | **Ecotype** | **Morphotype** |
| --- | --- | --- | --- |
| Aculeola_nigra | Squaliformes | benthopelagic | B |
| Alopias_pelagicus | Lamniformes | pelagic | A1 |
| Alopias_superciliosus | Lamniformes | pelagic | A1 |
| Alopias_vulpinus | Lamniformes | pelagic | A1 |
| Apristurus_albisoma | Carcharhiniformes | benthic | A1 |
| Apristurus_aphyodes | Carcharhiniformes | benthic | A1 |
| Apristurus_australis | Carcharhiniformes | benthic | A1 |
| Apristurus_brunneus | Carcharhiniformes | benthopelagic | A1 |
| Apristurus_bucephalus | Carcharhiniformes | benthic | A1 |
| Apristurus_canutus | Carcharhiniformes | benthic | A1 |
| Apristurus_exsanguis | Carcharhiniformes | benthic | A1 |
| Apristurus_fedorovi | Carcharhiniformes | benthic | A1 |
| Apristurus_gibbosus | Carcharhiniformes | benthic | A1 |
| Apristurus_herklotsi | Carcharhiniformes | benthic | A1 |
| Apristurus_indicus | Carcharhiniformes | benthic | A1 |
| Apristurus_internatus | Carcharhiniformes | benthic | A1 |
| Apristurus_investigatoris | Carcharhiniformes | benthic | A1 |
| Apristurus_japonicus | Carcharhiniformes | benthopelagic | A1 |
| Apristurus_kampae | Carcharhiniformes | benthic | A1 |
| Apristurus_laurussonii | Carcharhiniformes | benthopelagic | A1 |
| Apristurus_longicephalus | Carcharhiniformes | benthic | A1 |
| Apristurus_macrorhynchus | Carcharhiniformes | benthic | A1 |
| Apristurus_macrostomus | Carcharhiniformes | benthic | A1 |
| Apristurus_melanoasper | Carcharhiniformes | benthic | A1 |
| Apristurus_microps | Carcharhiniformes | benthopelagic | A1 |
| Apristurus_micropterygeus | Carcharhiniformes | benthic | A1 |
| Apristurus_nasutus | Carcharhiniformes | benthopelagic | A2 |
| Apristurus_parvipinnis | Carcharhiniformes | benthopelagic | A1 |
| Apristurus_pinguis | Carcharhiniformes | benthic | A1 |
| Apristurus_platyrhynchus | Carcharhiniformes | benthic | A1 |
| Apristurus_profundorum | Carcharhiniformes | benthic | A1 |
| Apristurus_riveri | Carcharhiniformes | benthic | A1 |
| Apristurus_saldanha | Carcharhiniformes | benthic | A1 |
| Apristurus_sibogae | Carcharhiniformes | benthic | A1 |
| Apristurus_sinensis | Carcharhiniformes | benthic | A1 |
| Apristurus_spongiceps | Carcharhiniformes | benthopelagic | A1 |
| Apristurus_stenseni | Carcharhiniformes | benthic | A1 |
| Asymbolus_analis | Carcharhiniformes | benthic | A2 |
| Asymbolus_funebris | Carcharhiniformes | benthic | A2 |
| Asymbolus_galacticus | Carcharhiniformes | benthic | A2 |
| Asymbolus_occiduus | Carcharhiniformes | benthic | A2 |
| Asymbolus_pallidus | Carcharhiniformes | benthic | A2 |
| Asymbolus_parvus | Carcharhiniformes | benthic | A2 |
| Asymbolus_rubiginosus | Carcharhiniformes | benthic | A2 |
| Asymbolus_submaculatus | Carcharhiniformes | benthic | A2 |
| Asymbolus_vincenti | Carcharhiniformes | benthic | A2 |
| Atelomycterus_baliensis | Carcharhiniformes | benthic | A2 |
| Atelomycterus_fasciatus | Carcharhiniformes | benthic | A2 |
| Atelomycterus_macleayi | Carcharhiniformes | benthic | A2 |
| Atelomycterus_marnkalha | Carcharhiniformes | benthic | A2 |
| Aulohalaelurus_kanakorum | Carcharhiniformes | benthic | A2 |
| Aulohalaelurus_labiosus | Carcharhiniformes | benthic | A2 |
| Brachaelurus_colcloughi | Orectolobiformes | benthic | A2 |
| Brachaelurus_waddi | Orectolobiformes | benthic | A2 |
| Bythaelurus_canescens | Carcharhiniformes | benthic | A2 |
| Bythaelurus_clevai | Carcharhiniformes | benthic | A2 |
| Bythaelurus_dawsoni | Carcharhiniformes | benthic | A2 |
| Bythaelurus_giddingsi | Carcharhiniformes | benthic | A2 |
| Bythaelurus_hispidus | Carcharhiniformes | benthic | A2 |
| Bythaelurus_immaculatus | Carcharhiniformes | benthic | A2 |
| Bythaelurus_incanus | Carcharhiniformes | benthic | A2 |
| Bythaelurus_lutarius | Carcharhiniformes | benthic | A2 |
| Carcharhinus_acronotus | Carcharhiniformes | pelagic | B |
| Carcharhinus_albimarginatus | Carcharhiniformes | pelagic | B |
| Carcharhinus_altimus | Carcharhiniformes | pelagic | B |
| Carcharhinus_amblyrhynchoides | Carcharhiniformes | pelagic | B |
| Carcharhinus_amblyrhynchos | Carcharhiniformes | pelagic | B |
| Carcharhinus_amboinensis | Carcharhiniformes | pelagic | B |
| Carcharhinus_borneensis | Carcharhiniformes | pelagic | B |
| Carcharhinus_brachyurus | Carcharhiniformes | pelagic | B |
| Carcharhinus_brevipinna | Carcharhiniformes | pelagic | B |
| Carcharhinus_cautus | Carcharhiniformes | pelagic | B |
| Carcharhinus_coatesi | Carcharhiniformes | pelagic | B |
| Carcharhinus_dussumieri | Carcharhiniformes | pelagic | B |
| Carcharhinus_falciformis | Carcharhiniformes | pelagic | B |
| Carcharhinus_fitzroyensis | Carcharhiniformes | pelagic | B |
| Carcharhinus_galapagensis | Carcharhiniformes | pelagic | B |
| Carcharhinus_hemiodon | Carcharhiniformes | pelagic | B |
| Carcharhinus_isodon | Carcharhiniformes | pelagic | B |
| Carcharhinus_leiodon | Carcharhiniformes | pelagic | B |
| Carcharhinus_leucas | Carcharhiniformes | pelagic | B |
| Carcharhinus_limbatus | Carcharhiniformes | pelagic | B |
| Carcharhinus_longimanus | Carcharhiniformes | pelagic | B |
| Carcharhinus_macloti | Carcharhiniformes | pelagic | B |
| Carcharhinus_melanopterus | Carcharhiniformes | pelagic | B |
| Carcharhinus_obscurus | Carcharhiniformes | pelagic | B |
| Carcharhinus_perezii | Carcharhiniformes | benthic | B |
| Carcharhinus_plumbeus | Carcharhiniformes | pelagic | B |
| Carcharhinus_porosus | Carcharhiniformes | pelagic | B |
| Carcharhinus_sealei | Carcharhiniformes | pelagic | B |
| Carcharhinus_signatus | Carcharhiniformes | pelagic | B |
| Carcharhinus_sorrah | Carcharhiniformes | pelagic | B |
| Carcharhinus_tilstoni | Carcharhiniformes | pelagic | B |
| Carcharhinus_tjutjot | Carcharhiniformes | pelagic | B |
| Carcharias_taurus | Lamniformes | benthopelagic | A1 |
| Carcharodon_carcharias | Lamniformes | pelagic | B |
| Centrophorus_atromarginatus | Squaliformes | benthic | B |
| Centrophorus_granulosus | Squaliformes | benthopelagic | B |
| Centrophorus_harrissoni | Squaliformes | benthic | B |
| Centrophorus_isodon | Squaliformes | benthic | B |
| Centrophorus_lusitanicus | Squaliformes | benthic | B |
| Centrophorus_moluccensis | Squaliformes | benthopelagic | B |
| Centrophorus_seychellorum | Squaliformes | benthic | B |
| Centrophorus_squamosus | Squaliformes | benthopelagic | B |
| Centrophorus_tessellatus | Squaliformes | benthic | B |
| Centrophorus_westraliensis | Squaliformes | benthopelagic | B |
| Centrophorus_zeehaani | Squaliformes | benthopelagic | B |
| Centroscyllium_excelsum | Squaliformes | benthic | B |
| Centroscyllium_fabricii | Squaliformes | benthic | B |
| Centroscyllium_granulatum | Squaliformes | benthic | B |
| Centroscyllium_kamoharai | Squaliformes | benthic | B |
| Centroscyllium_nigrum | Squaliformes | benthopelagic | B |
| Centroscyllium_ornatum | Squaliformes | benthic | B |
| Centroscyllium_ritteri | Squaliformes | benthic | B |
| Centroscymnus_coelolepis | Squaliformes | benthic | B |
| Centroselachus_crepidater | Squaliformes | benthopelagic | B |
| Cephaloscyllium_albipinnum | Carcharhiniformes | benthic | A1 |
| Cephaloscyllium_cooki | Carcharhiniformes | benthic | A2 |
| Cephaloscyllium_fasciatum | Carcharhiniformes | benthic | A1 |
| Cephaloscyllium_hiscosellum | Carcharhiniformes | benthic | A2 |
| Cephaloscyllium_isabellum | Carcharhiniformes | benthic | A2 |
| Cephaloscyllium_laticeps | Carcharhiniformes | benthic | A2 |
| Cephaloscyllium_maculatum | Carcharhiniformes | benthic | A2 |
| Cephaloscyllium_pardelotum | Carcharhiniformes | benthic | A1 |
| Cephaloscyllium_pictum | Carcharhiniformes | benthic | A2 |
| Cephaloscyllium_sarawakensis | Carcharhiniformes | benthic | A1 |
| Cephaloscyllium_signourum | Carcharhiniformes | benthic | A2 |
| Cephaloscyllium_silasi | Carcharhiniformes | benthic | A2 |
| Cephaloscyllium_speccum | Carcharhiniformes | benthic | A2 |
| Cephaloscyllium_stevensi | Carcharhiniformes | benthic | A2 |
| Cephaloscyllium_sufflans | Carcharhiniformes | benthic | A2 |
| Cephaloscyllium_umbratile | Carcharhiniformes | benthic | A1 |
| Cephaloscyllium_variegatum | Carcharhiniformes | benthic | A2 |
| Cephaloscyllium_ventriosum | Carcharhiniformes | benthic | A2 |
| Cephaloscyllium_zebrum | Carcharhiniformes | benthic | A2 |
| Cephalurus_cephalus | Carcharhiniformes | benthic | A1 |
| Cetorhinus_maximus | Lamniformes | pelagic | B |
| Chaenogaleus_macrostoma | Carcharhiniformes | benthic | B |
| Chiloscyllium_arabicum | Orectolobiformes | benthic | A2 |
| Chiloscyllium_burmensis | Orectolobiformes | benthic | A2 |
| Chiloscyllium_griseum | Orectolobiformes | benthic | A1 |
| Chiloscyllium_hasseltii | Orectolobiformes | benthic | A2 |
| Chiloscyllium_indicum | Orectolobiformes | benthic | A2 |
| Chiloscyllium_plagiosum | Orectolobiformes | benthic | A2 |
| Chiloscyllium_punctatum | Orectolobiformes | benthic | A2 |
| Chlamydoselachus_africana | Hexanchiformes | benthopelagic | A1 |
| Chlamydoselachus_anguineus | Hexanchiformes | benthopelagic | A1 |
| Cirrhigaleus_barbifer | Squaliformes | benthopelagic | B |
| Cirrhoscyllium_expolitum | Orectolobiformes | benthic | A2 |
| Cirrhoscyllium_formosanum | Orectolobiformes | benthic | A2 |
| Cirrhoscyllium_japonicum | Orectolobiformes | benthic | A2 |
| Ctenacis_fehlmanni | Carcharhiniformes | benthic | B |
| Dalatias_licha | Squaliformes | benthopelagic | B |
| Deania_calcea | Squaliformes | benthic | B |
| Deania_hystricosa | Squaliformes | benthic | B |
| Deania_profundorum | Squaliformes | benthic | B |
| Deania_quadrispinosa | Squaliformes | benthic | B |
| Echinorhinus_brucus | Echinorhiniformes | benthopelagic | A1 |
| Echinorhinus_cookei | Echinorhiniformes | benthopelagic | A1 |
| Eridacnis_barbouri | Carcharhiniformes | benthic | A2 |
| Eridacnis_radcliffei | Carcharhiniformes | benthic | A2 |
| Eridacnis_sinuans | Carcharhiniformes | benthic | A2 |
| Etmopterus_bigelowi | Squaliformes | benthopelagic | B |
| Etmopterus_brachyurus | Squaliformes | benthopelagic | B |
| Etmopterus_bullisi | Squaliformes | benthic | B |
| Etmopterus_burgessi | Squaliformes | benthic | B |
| Etmopterus_carteri | Squaliformes | benthic | B |
| Etmopterus_caudistigmus | Squaliformes | benthic | B |
| Etmopterus_compagnoi | Squaliformes | benthic | B |
| Etmopterus_decacuspidatus | Squaliformes | benthic | B |
| Etmopterus_evansi | Squaliformes | benthic | B |
| Etmopterus_fusus | Squaliformes | benthic | B |
| Etmopterus_gracilispinis | Squaliformes | benthopelagic | B |
| Etmopterus_granulosus | Squaliformes | benthic | B |
| Etmopterus_hillianus | Squaliformes | benthopelagic | B |
| Etmopterus_joungi | Squaliformes | benthic | B |
| Etmopterus_litvinovi | Squaliformes | benthopelagic | B |
| Etmopterus_lucifer | Squaliformes | benthopelagic | B |
| Etmopterus_molleri | Squaliformes | benthic | B |
| Etmopterus_perryi | Squaliformes | benthic | B |
| Etmopterus_polli | Squaliformes | benthic | B |
| Etmopterus_princeps | Squaliformes | benthopelagic | B |
| Etmopterus_pseudosqualiolus | Squaliformes | benthopelagic | B |
| Etmopterus_pusillus | Squaliformes | benthopelagic | B |
| Etmopterus_pycnolepis | Squaliformes | benthopelagic | B |
| Etmopterus_robinsi | Squaliformes | benthic | B |
| Etmopterus_sculptus | Squaliformes | benthic | B |
| Etmopterus_sentosus | Squaliformes | benthic | B |
| Etmopterus_sheikoi | Squaliformes | benthic | B |
| Etmopterus_spinax | Squaliformes | benthic | B |
| Etmopterus_splendidus | Squaliformes | benthopelagic | B |
| Etmopterus_viator | Squaliformes | benthic | B |
| Etmopterus_villosus | Squaliformes | benthic | B |
| Etmopterus_virens | Squaliformes | benthic | B |
| Eucrossorhinus_dasypogon | Orectolobiformes | benthic | A1 |
| Euprotomicroides_zantedeschia | Orectolobiformes | | B |
| Figaro_boardmani | Carcharhiniformes | benthic | A2 |
| Figaro_striatus | Carcharhiniformes | benthic | A2 |
| Furgaleus_macki | Carcharhiniformes | benthopelagic | B |
| Galeocerdo_cuvier | Carcharhiniformes | pelagic | B |
| Galeorhinus_galeus | Carcharhiniformes | benthopelagic | B |
| Galeus_antillensis | Carcharhiniformes | benthopelagic | A2 |
| Galeus_arae | Carcharhiniformes | benthopelagic | A1 |
| Galeus_atlanticus | Carcharhiniformes | benthic | A1 |
| Galeus_cadenati | Carcharhiniformes | benthic | A1 |
| Galeus_eastmani | Carcharhiniformes | benthic | A1 |
| Galeus_gracilis | Carcharhiniformes | benthic | A2 |
| Galeus_longirostris | Carcharhiniformes | benthopelagic | A2 |
| Galeus_melastomus | Carcharhiniformes | benthic | A1 |
| Galeus_mincaronei | Carcharhiniformes | benthic | A2 |
| Galeus_murinus | Carcharhiniformes | benthic | A1 |
| Galeus_nipponensis | Carcharhiniformes | benthic | A2 |
| Galeus_piperatus | Carcharhiniformes | benthic | A1 |
| Galeus_polli | Carcharhiniformes | benthic | A1 |
| Galeus_priapus | Carcharhiniformes | benthic | A2 |
| Galeus_sauteri | Carcharhiniformes | benthic | A1 |
| Galeus_schultzi | Carcharhiniformes | benthic | A1 |
| Galeus_springeri | Carcharhiniformes | benthic | A2 |
| Ginglymostoma_cirratum | Orectolobiformes | benthopelagic | A1 |
| Glyphis_fowlerae | Carcharhiniformes | pelagic | B |
| Glyphis_gangeticus | Carcharhiniformes | benthic | B |
| Glyphis_glyphis | Carcharhiniformes | pelagic | B |
| Glyphis_siamensis | Carcharhiniformes | pelagic | B |
| Gogolia_filewoodi | Carcharhiniformes | benthopelagic | B |
| Gollum_attenuatus | Carcharhiniformes | benthic | B |
| Gollum_suluensis | Carcharhiniformes | benthic | B |
| Halaelurus_boesemani | Carcharhiniformes | benthic | A2 |
| Halaelurus_buergeri | Carcharhiniformes | benthic | A2 |
| Halaelurus_lineatus | Carcharhiniformes | benthic | A2 |
| Halaelurus_natalensis | Carcharhiniformes | benthic | A2 |
| Halaelurus_quagga | Carcharhiniformes | benthic | A2 |
| Halaelurus_sellus | Carcharhiniformes | benthic | A2 |
| Haploblepharus_edwardsii | Carcharhiniformes | benthic | A2 |
| Haploblepharus_fuscus | Carcharhiniformes | benthic | A2 |
| Haploblepharus_kistnasamyi | Carcharhiniformes | benthic | A2 |
| Hemigaleus_australiensis | Carcharhiniformes | benthopelagic | B |
| Hemigaleus_microstoma | Carcharhiniformes | benthopelagic | B |
| Hemipristis_elongata | Carcharhiniformes | pelagic | B |
| Hemiscyllium_freycineti | Orectolobiformes | benthic | A2 |
| Hemiscyllium_galei | Orectolobiformes | benthic | A2 |
| Hemiscyllium_hallstromi | Orectolobiformes | benthic | A2 |
| Hemiscyllium_henryi | Orectolobiformes | benthic | A2 |
| Hemiscyllium_michaeli | Orectolobiformes | benthic | A2 |
| Hemiscyllium_ocellatum | Orectolobiformes | benthic | A2 |
| Hemiscyllium_strahani | Orectolobiformes | benthic | A2 |
| Hemiscyllium_trispeculare | Orectolobiformes | benthic | A2 |
| Hemitriakis_abdita | Carcharhiniformes | benthic | B |
| Hemitriakis_complicofasciata | Carcharhiniformes | benthic | B |
| Hemitriakis_falcata | Carcharhiniformes | benthic | B |
| Hemitriakis_indroyonoi | Carcharhiniformes | benthic | B |
| Hemitriakis_japanica | Carcharhiniformes | benthic | B |
| Heptranchias_perlo | Hexanchiformes | benthopelagic | A1 |
| Heterodontus_francisci | Heterodontiformes | benthic | B |
| Heterodontus_galeatus | Heterodontiformes | benthic | B |
| Heterodontus_japonicus | Heterodontiformes | benthic | B |
| Heterodontus_mexicanus | Heterodontiformes | benthic | B |
| Heterodontus_omanensis | Heterodontiformes | benthic | B |
| Heterodontus_portusjacksoni | Heterodontiformes | benthic | B |
| Heterodontus_quoyi | Heterodontiformes | benthic | B |
| Heterodontus_ramalheira | Heterodontiformes | benthic | B |
| Heterodontus_zebra | Heterodontiformes | benthic | B |
| Heteroscymnoides_marleyi | Squaliformes | pelagic | B |
| Hexanchus_griseus | Hexanchiformes | benthic | A1 |
| Hexanchus_nakamurai | Hexanchiformes | benthic | A1 |
| Holohalaelurus_favus | Carcharhiniformes | benthic | A2 |
| Holohalaelurus_grennian | Carcharhiniformes | benthic | A2 |
| Holohalaelurus_melanostigma | Carcharhiniformes | benthic | A2 |
| Holohalaelurus_punctatus | Carcharhiniformes | benthic | A2 |
| Holohalaelurus_regani | Carcharhiniformes | benthic | A2 |
| Hypogaleus_hyugaensis | Carcharhiniformes | benthopelagic | B |
| Iago_garricki | Carcharhiniformes | benthic | B |
| Iago_omanensis | Carcharhiniformes | benthopelagic | B |
| Isistius_brasiliensis | Squaliformes | benthic | A2 |
| Isistius_labialis | Squaliformes | pelagic | A2 |
| Isistius_plutodus | Squaliformes | pelagic | A2 |
| Isogomphodon_oxyrhynchus | Carcharhiniformes | pelagic | B |
| Isurus_oxyrinchus | Lamniformes | pelagic | B |
| Isurus_paucus | Lamniformes | pelagic | B |
| Lamiopsis_temminckii | Carcharhiniformes | benthic | B |
| Lamiopsis_tephrodes | Carcharhiniformes | benthic | B |
| Lamna_ditropis | Lamniformes | pelagic | B |
| Lamna_nasus | Lamniformes | pelagic | B |
| Leptocharias_smithii | Carcharhiniformes | benthic | B |
| Megachasma_pelagios | Lamniformes | pelagic | A1 |
| Mitsukurina_owstoni | Lamniformes | pelagic | A1 |
| Mollisquama_parini | Squaliformes | benthic | B |
| Mustelus_albipinnis | Carcharhiniformes | benthic | B |
| Mustelus_antarcticus | Carcharhiniformes | benthopelagic | B |
| Mustelus_asterias | Carcharhiniformes | benthopelagic | B |
| Mustelus_californicus | Carcharhiniformes | benthopelagic | B |
| Mustelus_canis | Carcharhiniformes | benthopelagic | B |
| Mustelus_dorsalis | Carcharhiniformes | benthic | B |
| Mustelus_fasciatus | Carcharhiniformes | benthic | B |
| Mustelus_griseus | Carcharhiniformes | benthic | B |
| Mustelus_henlei | Carcharhiniformes | benthopelagic | B |
| Mustelus_higmani | Carcharhiniformes | benthic | B |
| Mustelus_lenticulatus | Carcharhiniformes | benthopelagic | B |
| Mustelus_lunulatus | Carcharhiniformes | benthic | B |
| Mustelus_manazo | Carcharhiniformes | benthic | B |
| Mustelus_mangalorensis | Carcharhiniformes | benthic | B |
| Mustelus_mento | Carcharhiniformes | benthic | B |
| Mustelus_minicanis | Carcharhiniformes | benthic | B |
| Mustelus_mustelus | Carcharhiniformes | benthopelagic | B |
| Mustelus_norrisi | Carcharhiniformes | benthic | B |
| Mustelus_palumbes | Carcharhiniformes | benthopelagic | B |
| Mustelus_punctulatus | Carcharhiniformes | benthic | B |
| Mustelus_ravidus | Carcharhiniformes | benthic | B |
| Mustelus_schmitti | Carcharhiniformes | benthopelagic | B |
| Mustelus_sinusmexicanus | Carcharhiniformes | benthic | B |
| Mustelus_stevensi | Carcharhiniformes | benthic | B |
| Mustelus_walkeri | Carcharhiniformes | benthic | B |
| Mustelus_whitneyi | Carcharhiniformes | benthic | B |
| Mustelus_widodoi | Carcharhiniformes | benthic | B |
| Nasolamia_velox | Carcharhiniformes | benthic | B |
| Nebrius_ferrugineus | Orectolobiformes | benthopelagic | A1 |
| Negaprion_acutidens | Carcharhiniformes | benthic | B |
| Negaprion_brevirostris | Carcharhiniformes | benthic | B |
| Notorynchus_cepedianus | Hexanchiformes | benthopelagic | A1 |
| Odontaspis_ferox | Lamniformes | benthopelagic | B |
| Odontaspis_noronhai | Lamniformes | benthopelagic | B |
| Orectolobus_halei | Orectolobiformes | benthic | A1 |
| Orectolobus_hutchinsi | Orectolobiformes | benthic | A2 |
| Orectolobus_japonicus | Orectolobiformes | benthic | A1 |
| Orectolobus_leptolineatus | Orectolobiformes | benthic | A2 |
| Orectolobus_maculatus | Orectolobiformes | benthic | A2 |
| Orectolobus_ornatus | Orectolobiformes | benthic | A1 |
| Orectolobus_parvimaculatus | Orectolobiformes | benthic | A2 |
| Orectolobus_reticulatus | Orectolobiformes | benthic | A1 |
| Orectolobus_wardi | Orectolobiformes | benthic | A1 |
| Oxynotus_bruniensis | Squaliformes | benthic | B |
| Oxynotus_caribbaeus | Squaliformes | benthic | B |
| Oxynotus_centrina | Squaliformes | benthic | B |
| Oxynotus_japonicus | Squaliformes | benthic | B |
| Oxynotus_paradoxus | Squaliformes | benthic | B |
| Paragaleus_leucolomatus | Carcharhiniformes | benthic | B |
| Paragaleus_pectoralis | Carcharhiniformes | benthic | B |
| Paragaleus_randalli | Carcharhiniformes | benthic | B |
| Paragaleus_tengi | Carcharhiniformes | benthic | B |
| Parascyllium_collare | Orectolobiformes | benthic | A2 |
| Parascyllium_elongatum | Orectolobiformes | benthic | A2 |
| Parascyllium_variolatum | Orectolobiformes | benthic | A2 |
| Parmaturus_albimarginatus | Carcharhiniformes | benthic | A2 |
| Parmaturus_bigus | Carcharhiniformes | benthic | A2 |
| Parmaturus_campechiensis | Carcharhiniformes | benthic | A1 |
| Parmaturus_lanatus | Carcharhiniformes | benthic | A2 |
| Parmaturus_macmillani | Carcharhiniformes | benthic | A2 |
| Parmaturus_melanobranchus | Carcharhiniformes | benthic | A2 |
| Parmaturus_pilosus | Carcharhiniformes | benthic | A1 |
| Parmaturus_xaniurus | Carcharhiniformes | benthic | A2 |
| Pentanchus_profundicolus | Carcharhiniformes | benthic | A1 |
| Planonasus_parini | Carcharhiniformes | benthic | B |
| Pliotrema_warreni | Pristiophoriformes | benthic | A2 |
| Poroderma_africanum | Carcharhiniformes | benthic | A2 |
| Poroderma_pantherinum | Carcharhiniformes | benthic | A2 |
| Prionace_glauca | Carcharhiniformes | pelagic | B |
| Pristiophorus_cirratus | Pristiophoriformes | benthic | A2 |
| Pristiophorus_delicatus | Pristiophoriformes | benthic | A2 |
| Pristiophorus_japonicus | Pristiophoriformes | benthic | A2 |
| Pristiophorus_nancyae | Pristiophoriformes | benthic | A2 |
| Pristiophorus_nudipinnis | Pristiophoriformes | benthic | A2 |
| Pristiophorus_schroederi | Pristiophoriformes | benthic | A2 |
| Proscyllium_habereri | Carcharhiniformes | benthic | B |
| Proscyllium_magnificum | Carcharhiniformes | benthic | B |
| Proscymnodon_macracanthus | Squaliformes | benthic | B |
| Proscymnodon_plunketi | Squaliformes | benthic | B |
| Pseudocarcharias_kamoharai | Lamniformes | pelagic | B |
| Pseudoginglymostoma_brevicaudatum | Orectolobiformes | benthic | A2 |
| Pseudotriakis_microdon | Carcharhiniformes | benthic | B |
| Rhincodon_typus | Orectolobiformes | pelagic | B |
| Rhizoprionodon_acutus | Carcharhiniformes | benthic | B |
| Rhizoprionodon_lalandii | Carcharhiniformes | benthic | B |
| Rhizoprionodon_longurio | Carcharhiniformes | benthic | B |
| Rhizoprionodon_oligolinx | Carcharhiniformes | benthic | B |
| Rhizoprionodon_porosus | Carcharhiniformes | benthic | B |
| Rhizoprionodon_taylori | Carcharhiniformes | benthic | B |
| Rhizoprionodon_terraenovae | Carcharhiniformes | benthic | B |
| Schroederichthys_bivius | Carcharhiniformes | benthic | A2 |
| Schroederichthys_chilensis | Carcharhiniformes | benthic | A2 |
| Schroederichthys_maculatus | Carcharhiniformes | benthic | A2 |
| Schroederichthys_saurisqualus | Carcharhiniformes | benthic | A2 |
| Schroederichthys_tenuis | Carcharhiniformes | benthic | A2 |
| Scoliodon_laticaudus | Carcharhiniformes | benthic | B |
| Scoliodon_macrorhynchos | Carcharhiniformes | benthic | B |
| Scyliorhinus_besnardi | Carcharhiniformes | benthic | A2 |
| Scyliorhinus_boa | Carcharhiniformes | benthic | A2 |
| Scyliorhinus_canicula | Carcharhiniformes | benthic | A2 |
| Scyliorhinus_capensis | Carcharhiniformes | benthic | A2 |
| Scyliorhinus_cervigoni | Carcharhiniformes | benthic | A2 |
| Scyliorhinus_comoroensis | Carcharhiniformes | benthic | A2 |
| Scyliorhinus_garmani | Carcharhiniformes | benthic | A2 |
| Scyliorhinus_haeckelii | Carcharhiniformes | benthic | A2 |
| Scyliorhinus_hesperius | Carcharhiniformes | benthic | A2 |
| Scyliorhinus_retifer | Carcharhiniformes | benthic | A2 |
| Scyliorhinus_stellaris | Carcharhiniformes | benthic | A2 |
| Scyliorhinus_tokubee | Carcharhiniformes | benthic | A2 |
| Scyliorhinus_torazame | Carcharhiniformes | benthic | A2 |
| Scylliogaleus_quecketti | Carcharhiniformes | benthic | B |
| Scymnodalatias_albicauda | Squaliformes | benthopelagic | B |
| Scymnodalatias_garricki | Squaliformes | benthopelagic | B |
| Scymnodalatias_oligodon | Squaliformes | pelagic | B |
| Scymnodalatias_sherwoodi | Squaliformes | benthic | B |
| Scymnodon_ringens | Squaliformes | benthopelagic | B |
| Somniosus_antarcticus | Squaliformes | pelagic | B |
| Somniosus_longus | Squaliformes | benthopelagic | B |
| Somniosus_microcephalus | Squaliformes | benthopelagic | B |
| Somniosus_pacificus | Squaliformes | benthic | B |
| Somniosus_rostratus | Squaliformes | benthopelagic | B |
| Sphyrna_corona | Carcharhiniformes | pelagic | B |
| Sphyrna_lewini | Carcharhiniformes | pelagic | B |
| Sphyrna_media | Carcharhiniformes | pelagic | B |
| Sphyrna_mokarran | Carcharhiniformes | pelagic | B |
| Sphyrna_tiburo | Carcharhiniformes | pelagic | B |
| Sphyrna_tudes | Carcharhiniformes | pelagic | B |
| Sphyrna_zygaena | Carcharhiniformes | pelagic | B |
| Squaliolus_aliae | Carcharhiniformes | pelagic | B |
| Squaliolus_laticaudus | Squaliformes | benthic | B |
| Squalus_acanthias | Squaliformes | benthopelagic | B |
| Squalus_albifrons | Squaliformes | benthic | B |
| Squalus_altipinnis | Squaliformes | benthic | B |
| Squalus_blainville | Squaliformes | benthopelagic | B |
| Squalus_brevirostris | Squaliformes | benthic | B |
| Squalus_bucephalus | Squaliformes | benthic | B |
| Squalus_chloroculus | Squaliformes | benthic | B |
| Squalus_crassispinus | Squaliformes | benthic | B |
| Squalus_cubensis | Squaliformes | benthopelagic | B |
| Squalus_edmundsi | Squaliformes | benthic | B |
| Squalus_formosus | Squaliformes | benthic | B |
| Squalus_grahami | Squaliformes | benthic | B |
| Squalus_hemipinnis | Squaliformes | benthic | B |
| Squalus_japonicus | Squaliformes | benthic | B |
| Squalus_lalannei | Squaliformes | benthic | B |
| Squalus_megalops | Squaliformes | benthopelagic | B |
| Squalus_melanurus | Squaliformes | benthopelagic | B |
| Squalus_mitsukurii | Squaliformes | benthopelagic | B |
| Squalus_montalbani | Squaliformes | benthic | B |
| Squalus_nasutus | Squaliformes | benthic | B |
| Squalus_notocaudatus | Squaliformes | benthic | B |
| Squalus_rancureli | Squaliformes | benthic | B |
| Squalus_raoulensis | Squaliformes | benthic | B |
| Squalus_suckleyi | Squaliformes | benthopelagic | B |
| Squatina_squatina | Squatiniformes | benthic | A2 |
| Stegostoma_fasciatum | Orectolobiformes | benthopelagic | A1 |
| Sutorectus_tentaculatus | Orectolobiformes | benthic | A1 |
| Triaenodon_obesus | Carcharhiniformes | benthic | B |
| Triakis_acutipinna | Carcharhiniformes | benthic | B |
| Triakis_maculata | Carcharhiniformes | benthic | B |
| Triakis_megalopterus | Carcharhiniformes | benthopelagic | B |
| Triakis_scyllium | Carcharhiniformes | benthopelagic | B |
| Triakis_semifasciata | Carcharhiniformes | benthopelagic | B |
| Trigonognathus_kabeyai | Squaliformes | benthic | B |
| Zameus_ichiharai | Squaliformes | benthopelagic | B |
| Zameus_squamulosus | Squaliformes | benthopelagic | B |
